# Roles of Non-Canonical Wnt Signalling Pathways in Bone Biology

**DOI:** 10.3390/ijms221910840

**Published:** 2021-10-07

**Authors:** Jasna Lojk, Janja Marc

**Affiliations:** 1 Department of Clinical Biochemistry, Faculty of Pharmacy, University of Ljubljana, 1000 Ljubljana, Slovenia; jasna.lojk@ffa.uni-lj.si; 2 University Clinical Center Ljubljana, Clinical Department of Clinical Chemistry and Biochemistry, 1000 Ljubljana, Slovenia

**Keywords:** non-canonical Wnt signalling pathway, bone mineral density, signalling crosstalk, osteogenesis

## Abstract

The Wnt signalling pathway is one of the central signalling pathways in bone development, homeostasis and regulation of bone mineral density. It consists of numerous Wnt ligands, receptors and co-receptors, which ensure tight spatiotemporal regulation of Wnt signalling pathway activity and thus tight regulation of bone tissue homeostasis. This enables maintenance of optimal mineral density, tissue healing and adaptation to changes in bone loading. While the role of the canonical/β-catenin Wnt signalling pathway in bone homeostasis is relatively well researched, Wnt ligands can also activate several non-canonical, β-catenin independent signalling pathways with important effects on bone tissue. In this review, we will provide a thorough overview of the current knowledge on different non-canonical Wnt signalling pathways involved in bone biology, focusing especially on the pathways that affect bone cell differentiation, maturation and function, processes involved in bone tissue structure regulation. We will describe the role of the two most known non-canonical pathways (Wnt/planar cell polarity pathways and Wnt/Ca^2+^ pathway), as well as other signalling pathways with a strong role in bone biology that communicate with the Wnt signalling pathway through non-canonical Wnt signalling. Our goal is to bring additional attention to these still not well researched but important pathways in the regulation of bone biology in the hope of prompting additional research in the area of non-canonical Wnt signalling pathways.

## 1. Introduction: The Role of Wnt Signalling Pathways in Bone Biology

The wingless-type mouse mammary tumour virus (MMTV) integration site family (Wnt) signalling pathway is a central and evolutionary conserved signalling pathway involved in several developmental and physiological processes in the organisms. It plays an essential role in embryonal development, the establishment of polarity axes and cell fate determinations, tissue and organ development, as well as tissue regeneration and repair in post-embryonal and adult life. On a cellular level, the pathway regulates different stages of cell differentiation and/or stem cell renewal, cell proliferation, cell adhesion, motility and apoptosis [1,2]. Due to its important roles, any dysregulation can lead to developmental defects or even embryonal death or can result in pleiotropic or age-related diseases in later life, including cancer [3], Alzheimer’s disease [4], metabolic disorders [5,6] and osteoporosis [7,8].

The involvement of the Wnt signalling pathway in bone biology and disease was first discovered through a mutation in the LRP5 co-receptor, which was associated with osteoporosis pseudoglioma syndrome (OPPG), an autosomal recessive disorder resulting in low bone mass, frequent fractures and bone deformations [9]. Since then, several other mutations in the components of the Wnt signalling pathways have been linked to various bone-related hereditary disorders [10], and the Wnt signalling pathway was recognised as one of the crucial pathways regulating bone development and formation. Moreover, several genome-wide association studies (GWAS) have linked single nucleotide polymorphisms in Wnt-related genes to age-related low bone mineral density (BMD) and fracture risk, shedding new light on the underlying processes of bone formation [11,12].

Wnt signalling pathways are in general divided into the canonical, β-catenin dependent Wnt signalling pathway, and non-canonical, β-catenin-independent signalling pathways, which are historically divided into Wnt/planar cell polarity (PCP) pathway and Wnt/Ca^2+^ pathway. In bone cells, the activity of the canonical Wnt signalling pathway generally leads to bone tissue formation and maintenance of bone mineral density in adults [13,14]. Canonical Wnt signalling promotes the commitment of mesenchymal stem cells (MSCs) to osteoblastic lineage and promotes differentiation and bone formation at the critical steps of osteoblast differentiation [15,16,17] while at the same time inhibiting osteoclast formation through osteoblast secreted factors [14,17,18]. On the other hand, osteoblast-derived Wnt5a activates the non-canonical Wnt signalling pathway in osteoclast precursors and stimulates their differentiation and consequent bone degradation [19]. Moreover, Wnt signalling pathways have been shown to crosstalk with several other signalling pathways, including receptor activator of NF-κB (RANK)/RANK ligand (RANKL)/NF-κB pathway, mechanistic target of rapamycin (mTOR) metabolic pathways, the mitogen-activated protein kinase (MAPK) pathway and Hippo signalling pathway, which are all tightly involved in bone physiology. These pathways either promote or inhibit each other on the transcriptional level or directly through interactions of the signal transduction pathways. Thus, although the canonical/β-catenin Wnt signalling pathway plays a crucial role in bone remodelling, it is not sufficient to explain the complex processes of regulation and communication between the main bone cell types (osteoblasts, osteoclast, osteocytes and osteal macrophages) as well as the cells in their immediate environment. The balanced activity of both canonical and non-canonical signals, as well as adequate crosstalk with other signalling pathways, are thus required for optimal bone homeostasis (i.e., balance between bone destruction mediated by osteoclasts and bone formation mediated by osteoblasts as part of continuous bone remodelling process).

In this review, we will explore the current knowledge on different non-canonical Wnt signalling pathways involved in bone biology, focusing especially on the pathways that affect bone cell differentiation, maturation and function, processes involved in bone tissue structure regulation. We will describe the signal transduction pathways and roles of Wnt/PCP and Wnt/Ca^2+^ pathways, as well as other signalling pathways that communicate with Wnt signalling pathway through non-canonical Wnt signalling. We will focus on pathways that are downstream of Wnt activation and signalling, such as RANK/RANKL NF-κB pathway with a crucial role in osteoclast differentiation, mTOR pathways important for adjustments of cell metabolism during cell differentiation, as well as the Hippo and MAPK signalling pathways, which seem to be required for a complete response to Wnt activation.

## 2. Overview of the Canonical and Non-Canonical Wnt Signalling Pathways

**Scheme 1:**
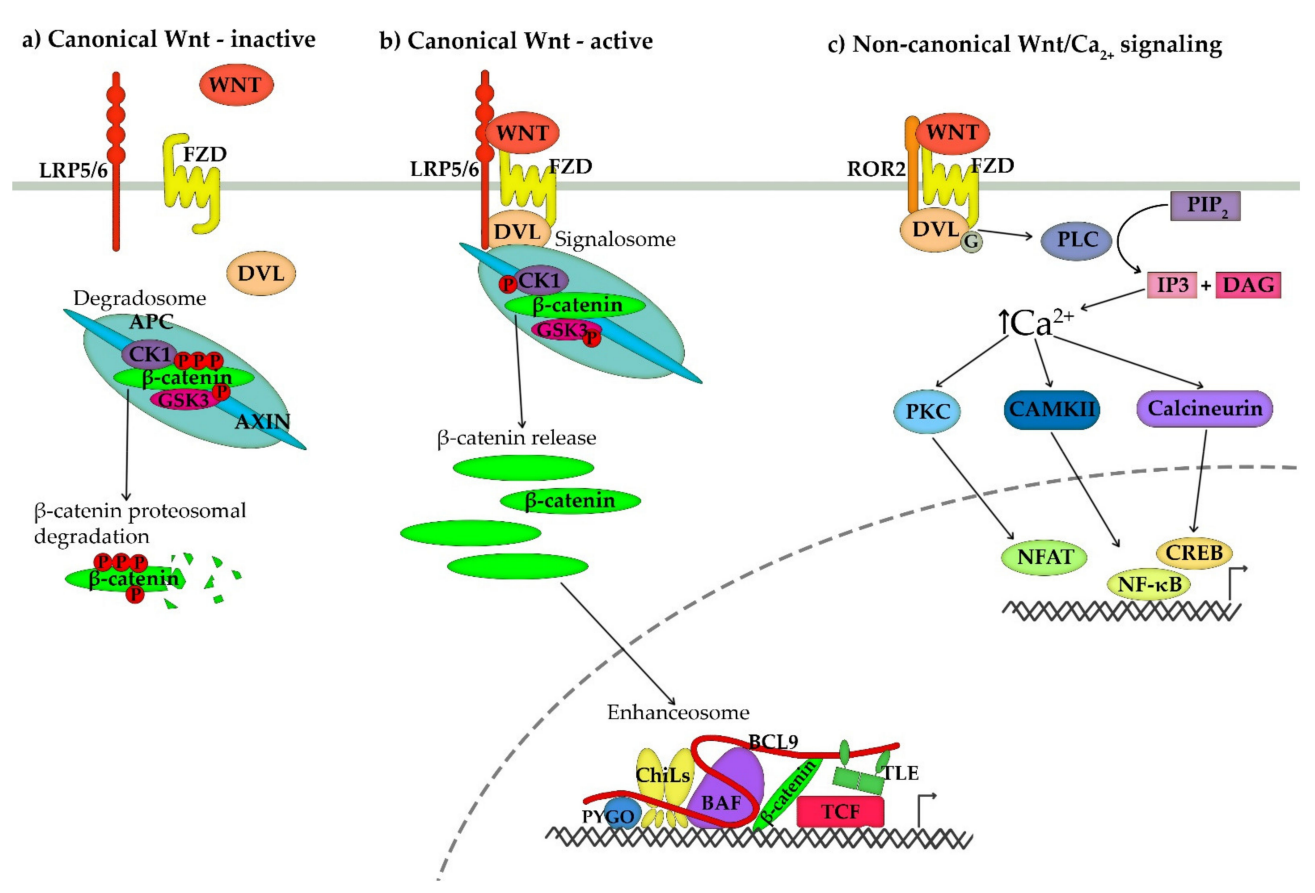
Schematic representation of (**a**) the inactive Wnt signalling pathway, (**b)** activated canonical Wnt signalling pathway and (**c**) non-canonical Wnt/Ca^2+^ signalling pathway. (**a**) In the absence of a Wnt ligand, the canonical Wnt signalling pathway is inactive, and β-catenin is degraded by a multiprotein complex termed axin degradosome. Degradosome is composed of scaffold proteins adenomatous polyposis coli (APC) and axin and kinases glycogen synthase kinase 3 (GSK3) and casein kinase 1 (CK1). The two kinases phosphorylate β-catenin and release it into the cytosol, where it is ubiquitinated and degraded by a proteasome complex. (**b**) Canonical Wnt signal transduction is initiated by the binding of Wnt ligand (WNT) to membrane frizzled (Fzd) receptor and low-density lipoprotein receptor-related protein 5/6 (LRP5/6) co-receptor. Fzd and LRP5/6 undergo dimerization and conformational changes, which recruit dishevelled (DVL) and degradosome to the membrane and trigger the assembly of a multiprotein complex termed signalosome. Inhibition of kinases releases β-catenin from the complex and enables its accumulation in the cytosol and translocation to the nucleus. In the nucleus, β-catenin incorporates into a multiprotein complex termed enhanceosome, composed of Pygopus (PYGO), ChiLs complex, adaptor protein B-cell lymphoma 9 (BCL9) and BAF (Brg/Brahma-associated factors) chromatin-remodelling complex. The main proteins of enhanceosome are transcriptional co-repressors TLE (transducin-like enhancer of split) and T cell factor (TCF) family of DNA-bound transcription factors, which mediate transcription of β-catenin dependent genes through changes in histone acetylation and chromatin condensation. (**c**) Non-canonical Wnt signalling is activated by binding a non-canonical Wnt ligand to Fzd receptor and Receptor tyrosine kinase-like orphan receptor 2 (ROR2). Receptor dimerization recruits DVL to the membrane and activates heterotrimeric G-proteins, which in turn activate phospholipase C (PLC). The PLC cleaves the membrane-bound phosphatidylinositol-4,5-bisphosphate (PIP2) into inositol-1,4,5-trisphosphate (IP3) and 1,2 diacylglycerol (DAG). IP3 induces the release of Ca^2+^ from intracellular calcium stores and activation of calcium-sensitive enzymes, such as protein kinase C (PKC), calmodulin-dependent protein kinase II (CaMKII) and calcineurin. These activated proteins, in turn, activate several transcription factors, such as nuclear factor κB (NF-κB), cAMP-responsive element-binding protein (CREB) and nuclear factor associated with T cells (NFAT).

The canonical Wnt/β-catenin signalling pathway is the most thoroughly researched Wnt signalling pathway, especially in relation to bone physiology. It is initiated by binding of a Wnt ligand to the frizzled (Fzd) receptor and two LDL receptor-related protein (LRP) co-receptors (LRP5/6), forming a tripartite complex [20]. This recruits the β-catenin destruction complex to the membrane (Scheme 1a). The destruction complex is a multiprotein complex responsible for the constant degradation of β-catenin, which prevents β-catenin accumulation in cytosol and translocation to the nucleus, where it could affect the transcription of Wnt target genes. Recruitment of the destruction complex to the membrane inhibits its degradation function and instead triggers the assembly of another multiprotein complex termed signalosome [21]. Signalosome enables the release of β-catenin and its translocation to the nucleus. There, β-catenin mediates gene transcription through its interaction with the third multiprotein complex termed enhanceosome [22]. Enhanceosome consists of several interacting proteins and protein complexes that mediate β-catenin loading into the complex, complex rearrangements, chromatin remodelling and transcription regulation (Scheme 1b). The main proteins of the enhanceosome are transcriptional co-repressors TLE (transducin-like enhancer of split) and T-cell factor/lymphoid enhancer factor (TCF/LEF) family of DNA-bound transcription factors [23,24]. These promote or repress transcription of Wnt response genes by regulating histone deacetylation and chromatin condensation [23,25].

In comparison, the non-canonical Wnt signalling pathway is a generic term that refers to signalling pathways triggered by Wnt ligands but not mediated by β-catenin. They are activated by specific Wnt ligands. However, due to the complexity, significant overlap and utilisation of the same core proteins and multitude of different outcomes, it is still not completely clear how many and which pathways there are. Two historically defined signalling pathways are Wnt/Ca^2+^ pathway, resulting in the release of intracellular calcium and activation of calcium-dependent processes, and the Wnt/PCP pathway, which leads to the establishment of cell polarity, changes in cytoskeleton arrangement, cell migration and attachments, although activation of several other transcription factors have also been observed [26], which will be discussed later.

Although the intracellular transduction pathways are markedly different, non-canonical and canonical Wnt signalling pathways share several extracellular and membrane components, especially Wnt ligands, certain receptors and Wnt endogenous inhibitors. In humans, 19 different Wnt ligands exist, and while certain Wnts activate only the canonical pathway, Wnt3a, Wnt4, Wnt5a, Wnt5b, Wnt7b and Wnt16 activate both the canonical and non-canonical pathways. Which pathway will be activated is most probably dependent on the quantity of Wnt ligands, expressed receptors, co-receptors and endogenous inhibitors [27,28,29,30]. Even Wnt5a, which is usually regarded as a non-canonical Wnt ligand, can, in the presence of Fzd4 and LRP5, activate the canonical Wnt signalling pathway [27,28]. Other ligands, such as Wnt3a, can activate both canonical and non-canonical signalling pathways in the same cell [29,31,32], and studies have shown that certain Wnt ligands can antagonise each other’s functions [33,34] or exert redundancy [35]. What effects certain Wnt ligands will have on cells also depends on the state of cell differentiation and the threshold levels of its activation [36].

Similar to the canonical Wnt signalling pathway, non-canonical signalling also requires an Fzd receptor. The frizzled protein family consists of 10 Fzd receptors, hepta-span transmembrane receptors, which can bind multiple Wnt ligands [37,38,39]. Not many studies have addressed the functional differences between the Fzd receptors, but substantial gene overlap and the presence of compensatory mechanisms indicate at least a partial functional redundancy [40,41]. However, instead of associating with LRP5/6 receptor, as required for canonical Wnt signalling, non-canonical Wnt signalling utilises alternative co-receptors, such as related to tyrosine kinase (Ryk), protein tyrosine kinase 7 (Ptk7) or receptor tyrosine kinase-like orphan receptor (ROR). Only ROR2 co-receptor has so far been shown to play an important role in bone biology and BMD regulation [19,42,43]. Similar to LRP5/6, it contains an extracellular cysteine-rich domain that resembles the Wnt binding site of Fzd receptors and an intracellular domain with tyrosine kinase activity, which is required for the transmission of the activation signal [44].

The last major component shared by canonical and non-canonical Wnt signalling pathways are endogenous Wnt inhibitors. While certain inhibitors, such as sclerostin (SOST) [45,46] and the Dickkopf WNT signalling pathway inhibitors (DKK) [47,48,49], can only inhibit LRP5/6 and Fzd functions, other inhibitors, such as Secreted frizzled-related proteins (sFRP) [50,51] and WNT inhibitory factor 1 (Wif1) [52], bind to the Wnt ligand directly and inhibit both canonical and non-canonical signalling. Their transcription and secretion can be triggered by both Wnt-related and -unrelated signals, which enable quick regulation of Wnt activities in relation to changes in external signals and establishment of gradients of signalling activity [53,54].

The large number of available ligands and receptors enable tight spatial and temporal regulation through ligand/receptor/co-receptor specificity, regulation of their transcription, as well as through secretion of endogenous Wnt inhibitors—the pathway will thus be activated only when all required molecules are present [55]. However, it is important to keep in mind that although these conditions can be achieved in vitro, such ligand, receptors and co-receptors combinations might not be available in vivo. The known effects of each Wnt ligand on bone cells have been summarised in Table 1.

**Table 1:** The effect of activation of the canonical and non-canonical signalling pathways with prominent roles in bone homeostasis. Only studies where the involved signalling pathway was confirmed are included.

Ligand	Effects of Activation of Non-Canonical	Non-Canonical Signal Transduction	Effects of Activation of Canonical
Wnt1	N/A		Stem cell maintenance [56]
			Promotion of Osteoblastogenesis [56,57]
Wnt2b	N/A		Osteogenic differentiation [58,59]
Wnt3a	Suppression of osteoclast differentiation	cAMP/PKA pathway [60]	Stem cell maintenance [36,61,62,63]
	Suppression of chondrocyte differentiation	c-Jun/AP-1 [60]	Suppression of osteoblastogenesis [64,65]
	Induction of osteoblastogenesis	PKCδ [66]	Suppression of osteoclastogenesis [60,67]
		mTORC1, mTORC2 [68,69]	Suppression of chondrogenesis [70]
		JNK pathway [31]	
	Promotion of osteoblastogenesis	RhoA [71]	
	Prevention of starvation induced apoptosis	c-Src/ERK1/2 and PI3K/Akt [32]	
Wnt4	Osteogenic differentiation, bone formation	p38 MAPK [72]	N/A
	Inhibition of osteoclast differentiation	TAK1-TAB2-NLK [73]	
Wnt5a	Promotion of osteoclastogenesis	DAAM2/Rho/PKN3/c-Src [74]	N/A
		JNK/c-Jun/Sp1 [19]	
		PKC/CAMKIIα/JNK [64]	
	Suppression of canonical Wnt signalling	PKC/CAMKIIα/JNK [64]	
		TAK1/NLK [75]	
	Prevention of starvation-induced apoptosis	c-Src/ERK1/2 and PI3K/Akt [32]	
	Promotion of chondrogenesis	CaMK/NFAT [76]	
	Inhibition of chondrogenesis	PI3K/Akt/IKK/NF-κB [76]	
	Promotion of osteoblastogenesis	RhoA [77]	
		JNK/c-Jun [78]	
Wnt5b	Suppression of chondrogenesis	JNK pathway [79]	N/A
Wnt7b	Promotion of osteoblastogenesis	mTORC1, mTORC2 [69,80]	N/A
		G_αq/11_/PLCβ/PKCδ [66]	
Wnt10b	Promotion of osteoblastogenesis	mTORC2 [69]	Promotion of osteoblastogenesis [81,82,83,84,85,86]
Wnt16	Suppression of osteoclastogenesis	JNK/c-Jun [86]	Suppression of osteoclastogenesis [87]
	Promotion of osteogenesis	JNK pathway [65]	

Abbreviations: Akt: protein kinase B; AP-1: activator protein 1; CAMK: calcium/calmodulin-dependent protein kinase; cAMP: cyclic adenosine monophosphate; c-Jun: Jun Proto-Oncogene, AP-1 transcription factor subunit; c-Src: SRC non-receptor tyrosine kinase; DAAM2: dishevelled associated activator of morphogenesis 2; ERK: extracellular signal-regulated kinase; IKK: inhibitor of nuclear factor-κB (IκB) kinase; JNK: c-Jun N-terminal kinase; MAPK: mitogen-activated protein kinase; mTORC: mechanistic target of rapamycin complex; NFAT: nuclear factor of activated T cells; NF-κB: nuclear factor κB; NLK: Serine/threonine-protein kinase NLK; PI3K: phosphatidylinositol 3-kinase; PLC: phospholipase C; PKA: protein kinase A; PKCδ: protein kinase C delta; PKN3: protein kinase N3; RhoA: Ras homolog family member A; TAB2: TGF-beta-activated kinase 1; TAK1: TGF-β–activated kinase 1.

## 3. Wnt/Ca^2+^ Non-Canonical Signalling Pathway

The Wnt/Ca^2+^ signalling pathway is a group of Wnt ligand-induced signalling pathways that result in an increase in intracellular Ca^2+^ concentration and the subsequent Ca^2+^-dependent cell signalling [87]. It regulates cytoskeletal rearrangements, cell attachment, migration [88] and differentiation and is involved in the establishment of dorsolateral asymmetry and somite patterning during embryonal development [89,90]. Calcium release is followed by the depolarisation of both the cell and nucleus membranes [91], and a study also suggested that a Wnt-induced increase in cytosolic Ca^2+^ concentration might promote canonical Wnt signalling by facilitating the transport of larger molecules (>10 kDa) across the nuclear envelope, thus increasing also the translocation of β-catenin [91].

Wnt5a and Wnt3a are considered prototypical activators of Wnt/Ca^2+^ signalling. They bind to ROR2 co-receptors and one of the Fzd receptors. For activation of calcium signalling, Wnt5a requires receptors Fzd2, 3, 4, 5 or 6 [89,92,93], out of which only Fzd4 [41,94,95] and Fzd6 [13,61,96] have so far been shown to play a role in bone biology. Following receptor activation, the pathway signals through heterotrimeric G-proteins, which leads to the activation of phospholipase C (PLC) [97,98,99]. The PLC cleaves the membrane-bound phosphatidylinositol-4,5-bisphosphate (PIP_2_) into inositol-1,4,5-trisphosphate (IP_3_) and 1,2 diacylglycerol (DAG). PI_3_ diffuses into the cytosol and binds to the IP3 receptors (IP3Rs), which function as calcium channels on the endoplasmatic reticulum, triggering the release of Ca^2+^ from intracellular stores [100]. Increased Ca^2+^ together with calmodulin, a phospho-serine/threonine-specific protein phosphatase, activates calcium-sensitive enzymes, such as calmodulin-dependent protein kinase II (CaMKII) and calcineurin [99]. DAG, on the other hand, together with Ca^2+^, activates protein kinase C (PKC) [89,99]. These activated proteins, in turn, activate several transcription factors, such as NF-κB, cAMP-responsive element-binding protein (CREB) and nuclear factor associated with T cells (NFAT).

The Wnt/Ca^2+^ signalling pathway is also required for osteoblast and osteoclast cell differentiation and function. In osteoblasts, Wnt3a and Wnt7b activate a Gα_q/11_/PLCβ/PKCδ pathway, through which they promote osteoblast differentiation and mineralisation in a β-catenin independent way. Inhibition of this pathway through KO of PKCδ or Wnt7b results in lower embryonal ossification, delayed chondrocyte maturation in long bones and reduced osteoblast differentiation [66]. Wnt/Ca^2+^ signalling also controls chondrogenesis. Bradley and Drissi have shown that Wnt5a promotes or inhibits chondrogenesis in a differentiation stage-specific manner. In the early stages, Wnt5a increases CaMK/NFAT and inhibits NF-κB-dependent signalling to promote chondrogenesis through an increase in SRY-Box transcription factor 9 (Sox9) expression. On the other hand, in later stages of differentiation, Wnt5a activates the PI3K/Akt/NF-κB signalling pathway and represses chondrocyte hypertrophy via NF-κB-dependent inhibition of RUNX family transcription factor 2 (Runx2) expression [76]. The two Wnt5a-induced signalling pathways thus inhibit each other in a stage-specific manner, which emphasises the importance of stage-dependent regulation and signalling in the control of cell differentiation.

Both canonical and non-canonical Wnt signalling pathways are also involved in Wnt suppression of RANKL induced osteoclast differentiation in osteoclast precursor cells. The non-canonical signalling is involved in the suppression of RANKL-mediated activation of the nuclear factor of activated T cells 1 (NFATc1) transcription factor. Wnt3a treatment led to a rapid increase in cyclic adenosine monophosphate (cAMP) accumulation, which induced phosphorylation and thus activation of protein kinase A (PKA), increasing phosphorylation (inactivation) of NFATc1 [60].

Interestingly, while there is ample evidence for tight cross-communication between osteoblast, osteocytes and osteoclast through both canonical and non-canonical Wnt signalling pathways, so far, no study has addressed the role of these pathways in communication with osteal macrophages (also osteomacs), resident macrophages in bone tissue. Even though osteal macrophages were shown to provide significant anabolic support during bone remodelling and fracture healing and are emerging as important players in bone homeostasis [101,102], there are still many open questions regarding their specific functional contributions. It would thus be interesting to explore whether the precise coordination between different bone cell types through the Wnt signalling pathway also includes osteal macrophages.

## 4. Wnt/Planar Cell Polarity (PCP) Pathway

**Scheme 2:**
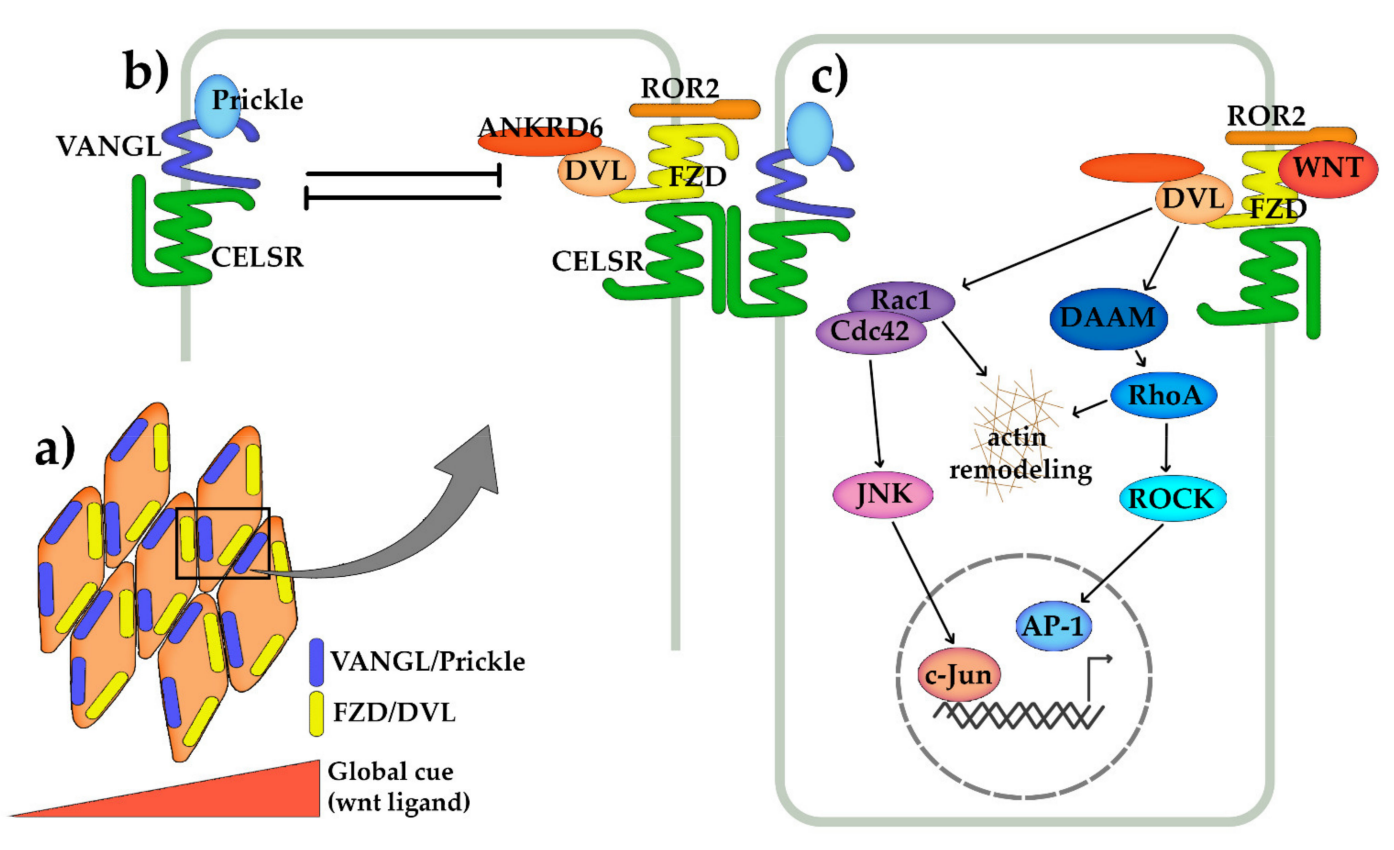
Schematic representation of Wnt/planar cell polarity (PCP) establishment, core components and signal transduction pathway. (**a**) Cell polarity is established in an epithelium based on the gradient of certain signalling molecules, such as Wnt5a ligands. It is mediated by two protein complexes that form on the opposite cell sites, and inter- and intracellular communication between these complexes provides the cells with a planar orientation axis pattern that can extend through the whole tissue. (**b**) The first complex is formed by a transmembrane frizzled receptor (Fzd) and cadherin EGF LAG seven-pass G-Type receptor (CELSR), which enable intercellular communication and cell–cell connection, and intracellular disheveled (DVL) and ankyrin repeat domain 6 (ANKRD6) proteins, which mediate intracellular signals. Similarly, the second complex is composed of transmembrane CELSR and Vangl planar cell polarity protein (VANGL) and the intracellular Prickle. The intracellular components are essential for negative feedback loops within each cell (they antagonise each other) and for activating signal transduction pathways. (**c**) Following Wnt ligand binding to Fzd receptor and ROR2 co-receptor, DVL is recruited to the membrane, which activates downstream signalling transductions. The activation signal is then transmitted to the adaptor protein dishevelled associated activator of morphogenesis (DAAM1-2) and small G proteins, such as Rac1, RhoA and Cdc42 as well as Rho-associated coiled kinase (ROCK1/2). The activation of GTPases triggers cytoskeleton rearrangements and activates transcriptions factors, such as c-jun NH2-terminal kinase (JNK) and activator protein1 (AP-1).

The PCP signalling pathway is a mechanism that provides global and local directional information that is required for cell and tissue polarisation, which is especially important during gastrulation and embryonal tissue formation. As such, it regulates cytoskeletal organisation, cell attachment and downstream cell signalling [103]. In vertebrates, the membrane-associated PCP core complex consists of six proteins that interact with each other inter- and intracellularly from the opposite sides of the cell. This provides cells with a planar orientation axis pattern that can extend through the whole tissue (Scheme 2a). The intercellular communication and connection are controlled by three transmembrane components of the complex; a Fzd receptor (particularly Fzd3 and Fzd6), Vangl planar cell polarity protein 2 (VANGL2) and cadherin EGF LAG seven-pass G-Type receptor (CELSR), while the intracellular signals are mediated through the tree cytoplasmic components disheveled (DVL), Prickle (PRICKLE) and ankyrin repeat domain 6 (ANKRD6) or Invesrin (INVS).

Through mutual inhibition, two complexes—Celsr-Vangl2-Prickle and Fzd-Celsr-DVL-Ankrd6—are established on the opposite sites of the cell [103,104,105]. The complex may also include additional components, such as Ryk and ROR2 co-receptors, which help relay the Wnt5a signal to Vangl2 to induce its phosphorylation [106,107,108,109]. Intracellularly, on the other hand, these two complexes stabilise each other and enable cell–cell communication required for the establishment of uniform PCP in a tissue [110,111] (Scheme 2b).

In bone tissue, the PCP signalling pathway and tissue polarisation have been associated with embryonal bone and joint formation, which involves cell migration, elongation and gradient-dependent differentiation [112]. For example, PCP is crucial during embryonal long bone cartilage elongation along the proximal–distal axis [113,114], which is mediated through the Wnt5a gradient in forming chondral tissue [115,116]. This gradient, in turn, induces a gradient in the Vangl2 phosphorylation end establishment of tissue polarity [106,117]. Similar effects were also observed for Wnt5b, where PCP activation was involved in Wnt5b-induced cell migration and chondrocyte differentiation [79]. The PCP pathway is also active postnatally, where it mediates orientation of cell division in strain-induced osteoblast proliferation, although proliferation itself is mediated through canonical Wnt signalling. Accordingly, mice with mutated Vangl2 exhibited altered bone architecture and a disorganised bone-forming surface [118]. The PCP signalling pathway was also shown to be involved in the migration and differentiation of osteoblast precursors in the frontal bone in mice [119].

The establishment of cell and tissue polarity is thus mainly involved in tissue organisation, growth and elongation, but not regulation of BMD. Instead, most effects of PCP signalling on osteoblast differentiation and function are attributed to changes in gene expression mediated by PCP pathway activation. It is not yet clear exactly how non-canonical Wnt ligands activate PCP-related signalling; however, several studies confirmed that Fzd receptor and ROR2 co-receptor are both required for Wnt/PCP signalling pathway activation, as is phosphorylation of Vangl [92,106,109]. The activation signal is then transmitted to the adaptor protein DAAM, small G proteins, such as Rac1, RhoA and Cdc42, and kinase Rho-associated coiled kinase (ROCK) [120] (Scheme 2c). Activation of Rho and ROCK triggers the assembly of stress fibres composed of contractile actin–myosin filaments and focal adhesion complexes [121,122], while activation of Rac promotes reassembly of actin filaments to form lamellipodia and membrane ruffles [123]. This induces changes in cell attachment and cytoskeletal organisation, which leads to cell migration and changes in cell polarity. Interestingly, cytoskeletal organisation and the subsequent RhoA and ROCK activity were also suggested to be the determining factor of MSC lineage commitment to osteo- or adipogenesis [77,122].

Apart from the cytoskeletal organisation, the Wnt/PCP signalling pathway can also activate the NH2-terminal kinase (JNK) and transcription factor c-jun, affecting downstream target genes [103,104,105]. JNK signalling, activated through Wnt3a or Wnt5a, seems to play an important role in the control of osteo- and the adipogenic fate of MSCs [31,71,78,124]. This balance between osteoblast and adipocyte commitment of MSCs, which might play a role in bone mass reduction during aging and osteoporosis [125], is also controlled through canonical and non-canonical RhoA/JNK Wnt signalling [31,71,77,78]. Similarly, canonical and non-canonical Wnt3a signalling inhibits chondrocyte differentiation and chondral matrix maturation through activation of the JNK signalling pathway and c-Jun and AP-1 transcription factors [70]. Osteoblast secreted Wnt5a can also enhance osteoclastogenesis through Wnt5a/ROR2 signalling, which increases expression of RANK in osteoclast precursor cells through activation of JNK/c-Jun/Sp1 [19] and promotes bone-resorbing osteoclast activity through DAAM2/Rho-protein kinase N3 (PKN3)/c-Src pathways [74]. The latter most probably regulates osteoclast function through the regulation of actin cytoskeleton, formation of stress fibres and actin rings, which are required for the formation of osteoclast resorption pit [126,127,128].

## 5. Non-Canonical Wnt Crosstalk with Other Signalling Pathways

The Wnt signalling pathway is part of an extensive and highly complex network of signalling pathways inside each cell. As such, the Wnt pathway can be stimulated or inhibited by other signalling pathways and vice versa; Wnt signalling activity can transactivate other signalling pathways [129]. In bone cells, Wnt was shown to interact with Bone morphogenic protein (BMP) signalling [130,131], the RANK/RANKL/NF-κB pathway, Hippo, Notch [132] and Hedgehog [133,134] signalling pathways, mTOR and epidermal growth factor (EGF) signalling [135] and most probably several others that have not been explored yet. Most of these interactions have been confirmed on the level of canonical Wnt signalling. In this chapter, we will focus on RANK/RANKL/NF-κB, mTOR, the Hippo and MAPK signalling pathways, which have been shown to be also regulated through non-canonical Wnt signalling.

### 5.1. Non-Canonical Wnt Crosstalk with RANKL/NF-κB Signalling Pathway

The RANK/RANKL signalling pathway is one of the main pathways regulating bone resorption. The receptor RANK is widely expressed in several cell types, including osteoclast precursor cells. It binds the RANK ligand (RANKL), which is in bone tissue secreted by mature osteoblasts, together with osteoprotegerin (OPG), a secreted non-signalling decoy receptor for RANKL that helps regulate osteoclast differentiation and function [136]. RANK/RANKL binding activates several signalling pathways, which lead to the activation of transcription factors essential for osteoclast activation, such as NF-κB, JNK/AP1 and NFAT1 [137]. Although the RANK/RANKL signalling axis and non-canonical WNT signalling share several signalling components and end effects, not many studies have shown a connection between the two systems. One mechanism of communication occurs through canonical Wnt signalling-mediated regulation of RANKL and OPG expression in osteoblasts, which was shown for Wnt3a, Wnt4 and Wnt16 [14,17,86,138,139,140]. Similarly, osteoblast-derived Wnt5a increases RANK expression in osteoclast precursor cells through Wnt5a/ROR2/JNK signalling by the recruitment of c-Jun transcription factor to RANK promoter, thus enhancing susceptibility of osteoclast precursor cells to osteoblast derived RANKL (Wnt5a itself failed to induce osteoclastogenesis in the absence of RANKL). Inhibition of this pathway leads to increased bone mass due to a reduction in bone resorption and osteoclast number [19]. Wnt5a induced RANK expression is thus required for adequate bone resorption.

**Scheme 3:**
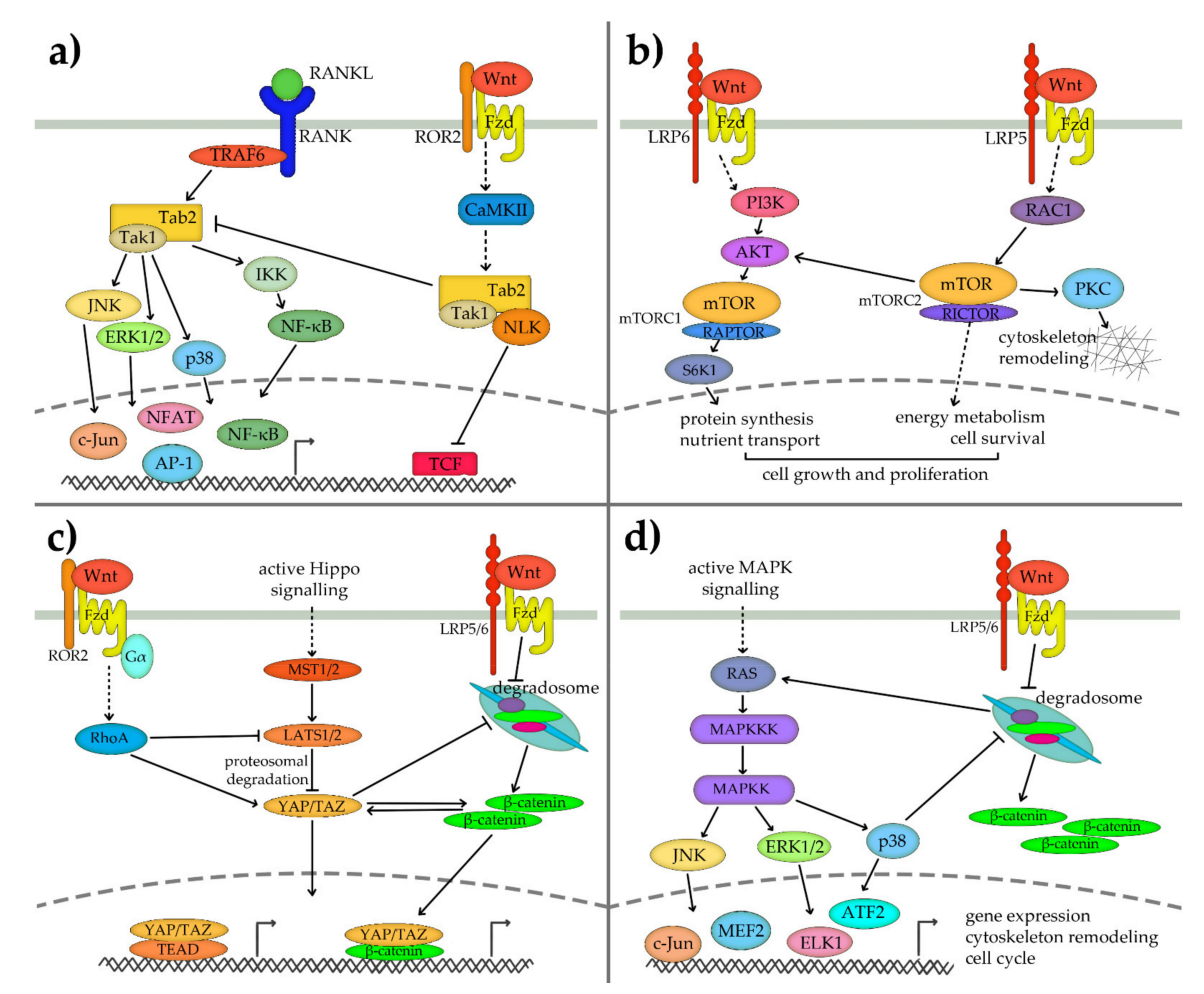
Schematic representation of cross-communication of non-canonical Wnt signalling pathways observed in bone cells. (**a**) Non-canonical Wnt signalling inhibits the RANK-RANKL signalling pathway through the inhibition of the formation of the TRAF6-TAB2-TAK1 complex by sequestering TAK1 to the Wnt-induced TAB2-TAK1-NLK complex. This complex activates NLK kinase, which inhibits the TCF/LEF protein family through phosphorylation. (**b**) The non-canonical Wnt signalling pathway interacts with mTOR in osteoblasts through two mechanisms. mTORC1 complex is activated through LRP6/Fzd-PI3K-AKT pathway and results in increased activity of S6 kinase 1 (S6K1) and increased protein synthesis. On the other hand, mTORC2 activation is mediated through Rho-family small GTPase RAC1 and leads to the activation of AKT and other signal transduction pathways, which results in changes in glucose and lipid energy metabolism, cell survival and cytoskeleton reorganisation. Both processes are required for adequate cell growth and proliferation. (**c**) The Hippo signalling pathway is activated through cell–cell attachment and cell adhesion. When active, it activates MST1/2 kinases and LATS1/2 kinases, which inhibit YAP/TAZ activity by inducing its proteosomal degradation. Non-canonical Wnt signalling through ROR2/Gα_12/13_ G protein/RhoA kinase inhibits YAP/TAZ inhibitor LATS1/2. This releases YAP/TAZ from degradation and enables it to translocate to the nucleus, where it forms complexes with different transcription factors, such as TEAD or β-catenin, to regulate transcription of downstream genes. YAP/TAZ also interacts with the canonical Wnt signalling pathway by blocking the degradosome function and promoting β-catenin cytoplasmic release. (**d**) MAPK signalling pathways involve the activation of small GTPases RAS and a cascade of sequentially activated kinases that lead to the activation of ERK1/2, JNK and p38 MAPKs. Activation of canonical Wnt signalling and inhibition of degradosome not only leads to the release of β-catenin but also stabilises RAS and thus activates the MAPK cascade. At the same time, p38 promotes Wnt/β-catenin signalling through the inhibition of the destruction complex. Not enough information is known to reliably construct the interactions of the non-canonical Wnt with MAPK signalling pathway in bone cells.

The communication between RANK/RANKL and the Wnt signalling pathway can also occur on the level of signalling transduction pathways. For example, Wnt4 inhibits osteoclast differentiation of primary bone marrow macrophages and RAW264.7 cells induced by RANKL by repressing NF-κB-dependent genes. This is achieved by Wnt4 promoting the formation of Tak1-Tab2-NLK complex, a MAPK-related pathway, that was also shown to inhibit the canonical Wnt signalling pathway by inhibiting the activity of TCF. In this pathway, transforming growth factor beta-activated kinase 1 (Tak1) kinase activates the Serine/threonine-protein Nemo like kinase (NLK), which, in turn, phosphorylates LEF1/TCF family proteins and prevents their binding to DNA [75,141]. However, in its interaction with the RANK/RANKL signalling pathway, the Wnt4-induced formation of Tak1-Tab2-NLK complex sequesters Tak1 and prevents the formation of RANKL-signalling-induced Traf6-Tak1-Tab2 complex (Scheme 3a). This not only reduces NF-κB signalling, but also partially inhibits phosphorylation of ERK, p38 and JNK induced by RANKL. In mice, administration of Wnt4 reduced and even reversed ovariectomy-induced bone loss [73].

### 5.2. Non-Canonical Wnt Crosstalk with mTOR Pathway

The Wnt signalling pathway has also been shown to interact with mTORC1 (mechanistic target of rapamycin complex 1) and mTORC2, the two complexes formed by mTOR. The two complexes have distinctive functions; while mTORC1 is involved in the regulation of protein synthesis, ribosomal biogenesis and nutrient transport, mTORC2 controls glucose and lipid metabolism, survival and cytoskeletal organisation [142,143]. mTOR activation and signalling have been implicated in bone biology in several in vivo studies, which implicated the activity of mTOR complexes in osteogenesis, skeletal growth and formation [80,144,145], chondral tissue development [145,146] and osteoarthritis [147] in mice. mTOR is involved in a Wnt-induced increase in aerobic glycolysis and protein synthesis in osteoblasts and chondrocytes during differentiation [146,148,149], and several studies have shown that adequate mTOR activity is required for normal cell differentiation and mineralisation [144,150,151]. On the other hand, mTOR activity decreases during osteoclastogenesis [152]. Some of the results are conflicting, which might be a consequence of opposing effects of mTOR signalling in different differentiation stages [153,154]. Interestingly, mTOR and DEP domain containing MTOR interacting protein (Deptor), important components of both mTORC1 and mTORC2 complexes, have also been associated with BMD in GWAS studies [155,156,157,158].

Esen and co-workers showed that Wnt3a/mTORC2 signalling could induce the Warburg effect in differentiating cells, a switch to glycolytic metabolism despite the presence of oxygen [69]. They detected an increase in levels of the key proteins in the glycolytic pathway, such as glucose transporter 1 (GLUT1), hexokinase II (HK2) enzyme that catalyses the first rate-limiting step of glucose catabolism, phosphofructokinase 1 (PFK1), 6-phosphofructo-2-kinase/fructose-2, 6-bisphosphatase 3 (PFKFB3), lactate dehydrogenase A (LDHA), which catalyses the conversion of pyruvate to lactate, as well as pyruvate dehydrogenase kinase 1 (PDK1) that inactivates the pyruvate dehydrogenase complex [69]. This shift in glucose metabolism leads to reduced levels of acetyl-CoA and histone acetylation, which changes gene transcription in favour of osteoblast differentiation [159]. Moreover, increased Wnt/mTORC1 signalling also leads to increased glutamine catabolism during osteoblast differentiation, which is required for increased energy and protein synthesis requirements [68]. In vivo, mTOR activity leads to increased osteoblast number and activity, which results in increased bone formation, higher bone mass and bone accrual [80]. This Wnt3a-, Wnt10b- or Wnt7b-induced activation of mTORC1 and subsequent changes in cell metabolism are mediated through LRP6/PI3K/AKT/S6K1 (S6 kinase 1) signalling [80,160]. Increased activity of S6K1 following mTORC1 activation leads to increased phosphorylation of ribosomal protein S6 and consequently increased markers of osteogenic differentiation (Runx2, alkaline phosphatase [ALP], osteocalcin, collagen 1 α1 [COL1A]) [160]. Interestingly, another study showed that mTORC2 signalling is mediated through LRP5 and activation of Rho-family small GTPase RAC1 [69], suggesting a separate mechanism of activation for each mTOR complex (Scheme 3b). The two complexes also seem to have distinctive roles in MSC lineage determination; while mTORC1 activity promotes osteogenic differentiation, mTORC2 is more effective in stimulating adipogenesis [161].

The mTORC1 and Wnt signalling pathways in osteoblasts also communicate in the opposite direction. mTORC1 activity has been shown to inhibit the Wnt signalling pathway activity by decreasing cell surface levels of Fzd receptors. It influences the association of DVL with clathrin AP-2 adaptor [162], which is essential for clathrin-mediated Fzd internalisation and subsequent downstream Wnt signal transduction [163].

### 5.3. Non-Canonical Wnt Crosstalk with Hippo Signalling Pathway

The role of the Hippo signalling pathway in bone biology has been recognised only in recent years. The pathway is involved in the regulation of cell proliferation and differentiation, especially in response to changes in cell adhesion and mechanical stress [164,165], but also responds to BMPs [166,167] and Wnts [168,169,170]. The Hippo signalling pathway signals through Yes-associated protein (YAP) and Transcriptional co-activator with PDZ binding motif (TAZ), which downstream interact with TEA domain (TEAD) containing family transcriptional factors to induce gene transcription [171]. YAP also acts as a co-regulator for other bone-related transcription factors, such as Runx2 [172], peroxisome proliferator-activated receptor-γ (PPARγ) [173] and β-catenin [168]. In bone, the pathway regulates the proliferation and differentiation of osteoblast progenitors, suppresses adipogenesis and in this way promotes bone mass formation [170,174]. Similarly, it regulates osteoclast proliferation, differentiation and apoptosis, mainly through inhibition of RANKL-induced NF-κB and calcium signalling [175].

The Wnt and Hippo signalling pathways are tightly connected and regulate each other’s activities. YAP/TAZ, the main effectors of the Hippo signalling pathway, regulates β-catenin levels and activity through physically interacting with β-catenin or DVL by blocking the activation of DVL and/or the cytoplasmic sequestration of β-catenin [174,176]. Other studies also suggest that YAP could directly bind to β-catenin in the nucleus to form a YAP/β-catenin/TCF transcriptional complex [177]. On the other hand, YAP and TAZ are suggested to be β-catenin downstream target genes [170], and the levels of TAZ are regulated by the β-catenin destruction complex [168,169].

Most of these interactions are mediated through the canonical Wnt signalling pathway; however, one study indicated that non-canonical Wnt ligands Wnt5a, Wnt5b, Wnt4 and Wnt3a are also strong activators of YAP/TAZ activity. The YAP/TAZ activation was independent of LRP5/6 co-receptors and β-catenin but required Fzd2 or Fzd5 receptor, ROR1 co-receptor and Gα_12/13_ G protein, which lead to downstream activation of RhoA and Rac kinases and subsequent inhibition of YAP/TAZ inhibitors Lats1/2 [178] (Scheme 3c). This study also showed that the activation of both the Wnt and YAP/TAZ is required for osteogenic differentiation of MSC cells and that the suppression of YAP/TAZ abolished WNT4-induced enhancement of osteoblast differentiation [178]. The two signalling pathways thus seem to function in unison and are both required for an adequate response to differentiation stimuli, which was so far attributed to the Wnt or Hippo signalling pathway only. Additional studies are thus necessary to better evaluate the contributions of each pathway and their crosstalk.

### 5.4. Non-Canonical Wnt Crosstalk with MAPK Pathway

The mitogen-activated protein kinase (MAPK) pathway is a cascade involving small GTPases Ras and a series of sequentially activated protein kinases (MAPKKK → MAPKK → MAPK), which lead to the activation of MAPK level kinases: extracellular signal-regulated kinases 1/2 (ERK1/2), JNK and p38 MAPK. The pathway is involved in responses to extracellular signals and affects skeletal development and bone homeostasis through regulation of osteoblast commitment and differentiation [179]. Both ERK and p38 MAPK are expressed during osteoblast differentiation and their signalling is required for osteogenic commitment and differentiation mediated by bone morphogenic protein 2 (BMP2) [180,181,182]. While ERK pathway is involved in the regulation of cell proliferation [183,184], p38 signalling is required for adequate ALP activity and mineralisation [183,185]. Similarly, ERK and p38 MAPK also have central roles in the promotion of chondrogenesis [186].

MAPK and Wnt signalling pathways mainly communicate through the β-catenin destruction complex of the canonical Wnt signalling pathway (Scheme 3d). Inhibition of the β-catenin destruction complex upon Wnt ligand stimulation not only stabilises β-catenin, but also leads to stabilisation of Ras and consequent activation of the downstream MAPK cascade [187]. At the same time, p38 promotes Wnt/β-catenin signalling through phosphorylation of LPR6, which is required for recruitment of the destruction complex to the membrane [188], and phosphorylates and inactivates glycogen synthase kinase 3β (GSK3β), allowing release and cytoplasmic accumulation of β-catenin [120]. The canonical Wnt and MAPK signalling pathways are thus primarily connected through shared functions of the β-catenin destruction complex.

Nevertheless, several studies indicate MAPK kinase activity can also be influenced through the non-canonical Wnt signalling pathway, which is especially important for the regulation of MSC function. For example, Wnt3a has been shown to induce both canonical and non-canonical Wnt signalling pathways [31,32], and while canonical Wnt signalling is primarily associated with osteogenic differentiation and renewal of MSCs, non-canonical Wnt signalling has been associated with cell survival and prevention of apoptosis. This is mediated through Src/ERK1/2 and PI3K/Akt signalling cascades, which are triggered independently of LRP5/6, and results in increased transcription of anti-apoptotic B-cell lymphoma 2 (Bcl-2) [32,189]. Similar effects were also shown for non-canonical Wnt5a signalling [32]. MAPK-mediated increased survival of osteoblasts may result in increased mature osteoblast number and prolonged activity, which was so far mainly attributed to the stimulatory effects of the canonical Wnt signalling pathway.

Another Wnt-MAPK crosstalk was reported by Chang and colleagues, who showed that non-canonical Wnt4 signalling promoted osteoblast differentiation from MSCs, increased mineralisation in vitro and stimulated bone formation in vivo. Observed p38 MAPK activation was dependent on axin but did not affect JNK activity [72]. Similarly, the Wnt4-induced p38 MAPK pathway has also been shown to be involved in melatonin-induced osteogenic differentiation and suppression of osteoclastogenesis [190,191,192]. Melatonin treatment increased Wnt4 synthesis through the ERK1/2-Pax2–Egr1 pathway, which in turn promoted osteoblast differentiation and function through both the canonical Wnt/β-catenin signalling (Wnt4-Fzd1/6-LRP5/6–β-catenin) and non-canonical Wnt4-Fzd2-JNK–p38 signalling pathways [193,194,195].

## 6. Syndromes and Osteoporosis Treatments Related to Non-Canonical Wnt Signalling Pathways

Most clinical research has been focused on canonical Wnt signalling in association with skeletal dysplasia’s, such as Osteogenesis Imperfecta type XV (mutations in Wnt1), Sclerosteosis and Van Buchem disease (mutations in SOST), osteoporosis pseudoglioma syndrome, juvenile osteoporosis (mutations in LRP5) and many more [10]. Up to now, only two inherited bone diseases have been associated with the non-canonical Wnt/PCP signalling pathway; a) autosomal recessive Robinow syndrome type 1 (ARRS1; OMIM 268310), which is characterised by short stature and upper limbs, brachydactyly, facial dysmorphisms and genital hypoplasia, and b) autosomal dominant Brachydactyly type B1 (OMIM 113000), one of the most severe types of brachydactyly, which can result in hypoplastic or absent distal phalanges and nails of hands and feet [196,197]. Both diseases are a result of a dysregulated PCP/Wnt signalling pathway as a consequence of gain or loss of function mutations in Wnt5a, ROR2 or Rac family small GTPase 3 (RAC3) [113,198,199]. This pathway is active, especially during embryonal tissue development and elongation, and any dysregulation results in limb deformities [113].

Out of endogenous Wnt inhibitors, only mutations in sFRPs, a family of Wnt ligand protein inhibitors, affect both canonical and non-canonical Wnt signalling. Loss of function mutations in sFRP4 were associated with Pyle disease (OMIM 265900), a rare recessive disorder resulting in metaphyseal widening of long bones, cortex thinning, increased trabecular bone, decreased BMD and a consequently increased bone fragility [200,201]. Cortical thinning was associated with decreased periosteal and endosteal bone formation and increased endocortical resorption in mice as a consequence of disrupted Wnt-BMP signalling crosstalk [200] and increased osteoclast differentiation. The latter was attributed to the lack of sFRP4-mediated inhibition of non-canonical Wnt5a/ROR2/JNK signalling otherwise required for suppression of osteoclast differentiation [202]. Possible treatment could thus involve inhibition of ROR2 signalling in order to compensate for the lack of sFRP4-mediated inhibition [202], which can, however, be a double-edged sword, as ROR2 activation is also required for osteoblast differentiation [42,77,78]. On the other hand, inhibition and not stimulation of sFRP1 was shown to promote bone formation ex vivo [203,204,205] and in vivo in mice [206], indicating a different mechanism of action for each sFRP inhibitor. Their use as therapeutics is further hindered by several reported actions unrelated to Wnt signalling and their role in the development of different tissues and pathological processes [207].

Much work thus remains to be done in clinical and basic research to better understand all connections between different canonical and non-canonical Wnt signalling pathways and better define Wnt ligand/receptor specificity and expression patterns, as non-canonical Wnt signalling pathways are emerging as an important support of canonical Wnt signalling and represent a potential target for the treatment of skeletal diseases. Unfortunately, Wnt signalling pathways are not crucial only for bone development, and any increase or decrease in signalling activity could result in adverse effects on other organ systems or cancer. The possible novel Wnt-based therapies would thus have to adjust the delivery system in order to limit the effects of the therapy to the target tissue, or target bone-specific protein targets, as was achieved with anti-SOST therapeutics. Despite that, even FDA-approved SOST inhibitor EVENITY comes with a warning for potential risk of myocardial infarction, stroke or cardiovascular death.

## 7. Conclusions

The Wnt signalling pathway has been shown to have an enormous impact on bone development and remodeling. Variations in several Wnt-related genomic regions have been associated with changes in BMD or different bone disorders, and changes in signalling activity can easily tip the balance between bone formation and bone resorption into a pathological state. Despite the fact that most studies primarily attribute this to the canonical Wnt signalling pathway, non-canonical Wnt signalling has been time and again shown to also have an important role in the regulation of this balance, either through its direct effects, through interactions with the canonical Wnt signalling pathway, or through crosstalk with other signalling pathways crucial for appropriate bone development. After all, Wnt signalling is just a part of a complex network of signal transduction events that occur during processes such as differentiation. Dysregulation of any of the connected pathways might thus propagate a change to the whole signalling network and induce effects that cannot be explained by the observed pathway only.

Thus, when researching the many roles of Wnt signalling pathways in bone biology, it is important to consider that Wnt ligands can not only activate canonical and non-canonical signalling at the same time, as was demonstrated for Wnt3a, but could also activate more than one non-canonical Wnt signalling pathway. Future studies exploring the effects of Wnt signalling pathways should thus consider analysing also the possible activation of other (non-canonical) Wnt signalling pathways or even strongly interconnected pathways, such as Hippo or MAPK signalling pathways. This will result in a better understanding of the importance and impact of each pathway’s crosstalk, shine more light on the roles of GWAS hits associated with BMD and possibly provide the groundwork for novel anabolic bone therapies targeting more than one signalling pathway.

## Data Availability

No new data were created or analyzed in this study. Data sharing is not applicable to this article.
